# Comparison of Different Liver Test Thresholds for Drug-Induced Liver Injury: Updated RUCAM versus Other Methods

**DOI:** 10.3389/fphar.2019.00816

**Published:** 2019-07-19

**Authors:** Hongyi Yang, Daihong Guo, Yuanjie Xu, Man Zhu, Chong Yao, Chao Chen, Wangping Jia

**Affiliations:** Department of Pharmaceutical Care, Chinese People’s Liberation Army General Hospital, Beijing, China

**Keywords:** drug-induced liver injury, thresholds comparison, fluoroquinolones, active surveillance, pharmacovigilance

## Abstract

According to the updated Roussel Uclaf Causality Assessment Method (RUCAM), drug-induced liver injury (DILI) is currently defined based on thresholds of alanine aminotransferase (ALT) levels above 5 × the upper limit of normal (ULN) and/or alkaline phosphatase (ALP) levels greater than 2 × the ULN. However, many parameters with different thresholds are also currently used in the clinic. We therefore performed a comparative analysis to evaluate which set of criteria was the most appropriate to detect DILI. We enrolled hospitalized patients who received fluoroquinolones to treat or prevent infections. Three liver test criteria were used to diagnose DILI in these patients. RUCAM criteria were defined as the gold standard, and the other two criteria were as follows: 1) ALT or aspartate aminotransferase (AST) levels greater than 5 × the ULN on two consecutive occasions and/or ALP levels greater than 2 × the ULN on two consecutive occasions [issued by DILI Network (DILIN)]; 2) ALT levels greater than 1 × the ULN on two consecutive occasions or ALT levels greater than 2 × the ULN [issued by the National Medical Products Administration (NMPA) of China]. We found that the RUCAM criteria resulted in 657 warnings, DILIN criteria resulted in 358, NMPA criteria resulted in 1,377, and the positive predictive value (PPV) were 9.74%, 10.89%, and 9.73% (*P* = 0.80), respectively. The levels of agreement of the DILIN and NMPA criteria with the RUCAM criteria were moderate, but the agreement between the DILIN criteria and NMPA criteria was poor. In conclusion, the NMPA criteria with relatively lax thresholds for the parameters require much more labor to determine the diagnosis, making them unsuitable for clinical practice. Conversely, the DILIN criteria employing stricter thresholds for the parameters were more effective but would miss some positive cases, and the cases it identified were usually quite serious, which is not conductive to early intervention. Therefore, we still recommend the use of the RUCAM criteria in clinical practice.

## Introduction

Drug-induced liver injury (DILI) is a frequent adverse drug reaction (ADR) that may present multiple clinical manifestations and is also one of the most frequent reasons for drug non-approval or withdrawal from market, as it leads to acute liver failure and even death (Iorga et al., 2017; Donato and Tolosa, 2019; Shen et al., 2019). Thus, it is very important to diagnose DILI promptly and accurately. Unfortunately, the mechanism underlying DILI is not well understood, and due to the lack of specific biomarkers, clinicians are only able to diagnose DILI *via* exclusion, as the presence of liver injury must first be confirmed and then all other suspected causes of the injury must be excluded before the relationship between the liver injury and a particular medicine is ultimately determined (Real et al., 2018). Some annoying problems have been noted during the clinical causality assessment of patients with suspected liver injury, such as alternative diagnoses and missed diagnoses. Thus, patients may not be provided the appropriate specific therapies in a timely manner (Teschke et al., 2013; Teschke et al., 2014).

These problems have prompted the development of a new causality assessment method that is widely known as the Roussel Uclaf Causality Assessment Method (RUCAM) (Danan and Teschke, 2016). According to the updated RUCAM, DILI is currently defined based on thresholds of alanine aminotransferase (ALT) levels greater than 5 × the upper limit of normal (ULN) and/or alkaline phosphatase (ALP) levels greater than 2 × the ULN. RUCAM is used as the gold standard in many studies (Danan and Teschke, 2016). However, many independent research institutions or projects have proposed some parameters with different thresholds that have not yet been validated. Few comparative studies have indicated which set of criteria is the most suitable for use in clinical practice and research. Therefore, based on data from the Adverse Drug Events Active Surveillance and Assessment System (ADE-ASAS) developed by the People’s Liberation Army ADR Monitoring Center, we compared different liver test criteria in the same population to determine which one is the most appropriate and to encourage health care providers to closely monitor this ADR.

## Materials and Methods

### Study Design and Data Source

A variety of drugs have been found to be associated with the occurrence of DILI, and it is difficult to monitor all of them simultaneously. Fluoroquinolones are one of the most widely prescribed antibiotics and have been used in clinical practice for more than 20 years. Despite their effectiveness, fluoroquinolones are also associated with some rare but serious reactions, including DILI. Many national drug administrations from various countries have issued restrictions on the use of these drugs because of DILI. Thus, we chose fluoroquinolones as the target drug and enrolled patients who were admitted to the Chinese People’s Liberation Army General Hospital and received fluoroquinolones to treat or prevent infections from January 2016 to December 2017. Patient data, including demographic characteristics, diagnosis, prescriptions, laboratory test results, and outcomes, were extracted from the hospital information system through the ADE-ASAS.

**Figure 1:**
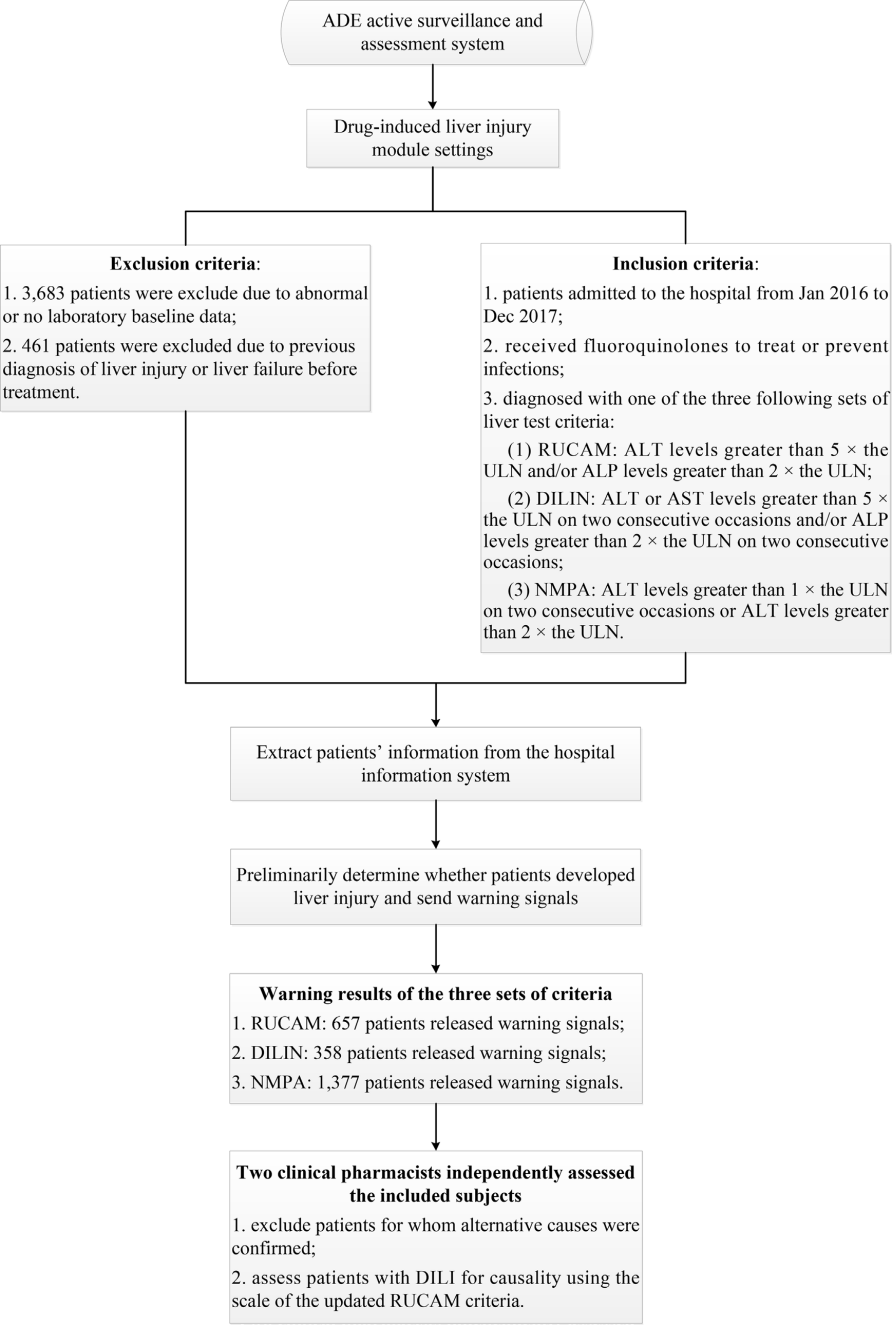
Flowchart of the assessment of positive cases.

The ADE-ASAS preliminarily identified suspected DILI cases and released warning signals once the monitoring indicators reached the criteria for DILI; then two clinical pharmacists conducted back-to-back evaluations to confirm the cases. If one patient had multiple positive signals during hospitalization, only the first signal was assessed. Detailed descriptions of the ADE-ASAS and its applications have been described in our previous study (Chen et al., 2018). In that study, we mainly aimed to obtain the incidence, clinical features, and to find out the risk factors of fluoroquinolone-induced liver injury, and we found out that many sets of criteria for diagnosis of DILI were used in various researches, and it makes sense to compare the differences among them. Thus, we conducted the present study with the same data source. The complete flowchart of the assessment is presented in **Figure 1**. The protocol of this study was approved by the Medical Ethics Committee of the Chinese People’s Liberation Army General Hospital and was conducted in accordance with the Declaration of Helsinki and the recommendations of the Medical Ethics Committee of the Chinese People’s Liberation Army General Hospital. Patient consent to review their medical records was not required because this was a retrospective study that did not infringe upon the interests or rights of the patients. All patient data were kept strictly confidential.

### Liver Test Criteria for DILI

The RUCAM was created in the late 1980s, and it is based on the results of international consensus meetings of experts, as documented by various reports and reviewed in recent studies (Danan and Teschke, 2016). The original version of the RUCAM has been constantly adjusted and modified, and it underwent some substantial improvements and clarifications of some core items, including time to onset from the beginning of the drug administration, course of ALT after cessation of the drug, risk factors, concomitant drugs, alternative causes, previous hepatotoxicity of the drug, and response to unintentional re-exposure. The sensitivity, specificity, and predictive values of the updated RUCAM have been validated (Danan and Teschke, 2016). Thus, we adopted the liver injury criteria prescribed by the RUCAM, ALT levels greater than 5 × the ULN and/or ALP levels greater than 2 × the ULN, and used it as the gold standard in this study.

We also identified many parameters with different thresholds that were used and reported in some articles and meetings, which mainly required increased liver enzyme levels in two consecutive measurements or lower thresholds (Molokhia and McKeigue, 2006; Lucena et al., 2011). For example, in terms of a causality assessment, the DILI Network (DILIN) method ignores missing data for important items and lacks a system that scores key elements, in contrast to the RUCAM. The liver injury criteria in the DILIN method are defined as ALT or aspartate aminotransferase (AST) levels greater than 5 × the ULN on two consecutive occasions and/or ALP levels greater than 2 × the ULN on two consecutive occasions and were also used in several studies (Dakhoul et al., 2018; Fontana et al., 2018; Cirulli et al., 2019). Moreover, the National Medical Products Administration (NMPA) of China has issued a set of criteria with lower thresholds that were defined as ALT levels greater than 1 × the ULN on two consecutive occasions or ALT levels greater than 2 × the ULN in one assessment, and these criteria are similar to another project called EUDRAGENE (Molokhia and McKeigue, 2006; Lucena et al., 2011). In addition to the RUCAM criteria, these two different criteria are the main additional standards used in the clinic, and their levels of strictness differ.

### System Settings and Manual Assessment

Patients with abnormal or no laboratory baseline data and patients who were diagnosed with liver injury or liver failure before treatment were automatically excluded by the ADE-ASAS. The inclusion criteria employed by the system were set according to the three sets of liver test criteria mentioned above, and the patients who met the criteria would prompt the system to send out warning signals. Two clinical pharmacists independently assessed the included subjects who released warning signals and excluded patients with other confirmed alternative causes, such as chronic liver diseases or biliary diseases. The RUCAM scale was used to evaluate the causal relationship between the drug and DILI. Patients with total scores greater than or equal to 6 (probable) were finally enrolled (Danan and Teschke, 2016; Teschke and Danan, 2018).

According to Hy’s law, we defined severe cases as follows: DILI resulting in increased ALT or AST levels greater than 3 × the ULN and total bilirubin levels greater than 2 × the ULN with no evidence of intra- or extra-hepatic bilirubin obstruction (elevated ALP levels) or Gilbert’s syndrome (Temple, 2006).

### Statistical Analysis

The normal distribution of continuous data was verified, and these data are described as the means ± standard deviation. Categorical data are described as the frequencies (constituent ratios). Between-group differences were analyzed using Student’s *t* tests or one-way ANOVA for continuous data and the chi-square test or Fisher’s exact test for categorical data. The positive predictive value (PPV) of each set of criteria was calculated as “positive cases/cases with warnings × 100%.” Partitioning of the chi-square test was used for pairwise comparisons. The *Kappa* coefficient was calculated to determine the level of agreement between the three sets of criteria. The thresholds of *Kappa* values utilized for interpretation were as follows: less than 0.40 was poor; 0.41 to 0.60 was moderate; 0.61 to 0.80 was good; and 0.81 to 1.00 was very good (Heidar et al., 2017).

All statistical analyses were performed with SPSS software, version 19.0 (SPSS Inc., Chicago, IL, USA). A two-sided *P* value less than 0.05 was set as the threshold for statistical significance.

## Results

### ADE-ASAS Monitoring Results

We identified 17,822 patients who received fluoroquinolones to prevent or treat infections, 4,144 of whom were excluded due to abnormal or a lack of baseline laboratory data or were diagnosed with liver injury or liver failure before treatment, and 13,678 patients were eventually enrolled. For these patients, the RUCAM criteria (ALT levels greater than 5 × the ULN and/or ALP levels greater than 2 × the ULN) released 657 (4.80%) warning signals, the DILIN criteria (ALT or AST levels greater than 5 × the ULN on two consecutive occasions and/or ALP levels greater than 2 × ULN on two consecutive occasions) released 358 (2.62%) warning signals, and the NMPA criteria (ALT levels greater than 1 × the ULN on two consecutive occasions or ALT levels greater than 2 × the ULN) released 1,377 (10.07%) warning signals. After manual assessment, patients with various liver or biliary diseases, such as hepatitis B, C, and E, CMV, EBV, ischemic hepatitis, cardiac hepatopathy, autoimmune hepatitis, non-alcoholic fatty liver disease, non-alcoholic steatohepatitis, and alcoholic liver disease, were also excluded. The case results that were confirmed positive are listed in **Table 1**. We only enrolled patients with probable or highly probable scores as positive cases, and no significant difference in the PPV was observed between the three sets of criteria (*P* = 0.80).

**Table 1:** Warnings and manual assessment results of the three sets of criteria.

	Warnings	Possible^1^	Positive Cases		Positive predictive value^4^
			Probable^2^	Highly probable^3^	
RUCAM criteria	657	27	53	11	9.74%
DILIN criteria	358	14	30	9	10.89%
NMPA criteria	1,377	75	116	18	9.73%

^1^The total score for the causality assessment was 3 to 5 points. ^2^The total score for the causality assessment was 6 to 8 points. ^3^The total score for the causality assessment was greater than 9 points. ^4^Calculated as “positive cases/cases with warnings × 100%.”

The demographic and clinical characteristics of the three groups of positive cases are presented in **Table 2**. There were no significant differences except for the pattern of liver injury (*P* < 0.01). According to manual assessment, we can find that there were more patients with hepatocellular injury in the RUCAM group and DILIN group, but the proportion of mixed injury was the largest in the NMPA group. Of the three groups, DILI occurred in about half of the patients between 5 and 90 days after the beginning of the drug, and it occurred in a few patients after cessation of therapy. Furthermore, combinations with other drugs known as hepatotoxin were commonly observed in three groups. Most of these concomitant drugs were other antibiotics and tuberculostatics, such as cephalosporins and isoniazide, and the scores of these patients were mainly distributed at 6 to 8 points. We did not observe the patients who were re-exposed to the drug because our target drug is a kind of antibiotic, which is generally used until the patient’s condition improves or it would be replaced if the treatment is not effective. Moreover, it is harmful to intentionally re-expose the patients to the suspect drug, and the risk to induce a more serious liver injury is high, and sometimes, the outcome is fatal (Danan and Teschke, 2016).

**Table 2:** Demographic and clinical characteristics of three groups of positive cases.

Variable	RUCAM criteria^1^ (*n* = 64)	DILIN criteria^2^ (*n* = 39)	NMPA criteria^3^ (*n* = 134)	*P* value
Age (years)	55.00 ± 18.35	59.79 ± 15.91	58.49 ± 17.88	0.32
Sex (% male)	47 (73.4)	27 (69.2)	95 (70.9)	0.89
Ethnicity				0.19
Han	63 (98.4)	37 (94.9)	123 (91.8)	
Non-Han	1 (1.6)	2 (5.1)	11 (8.2)	
BMI (kg/m^2^)	22.45 ± 3.85	21.95 ± 3.71	23.19 ± 3.84	0.16
Pattern of liver injury				<0.01
Hepatocellular	27 (42.2)	18 (46.2)	32 (23.9)	
Cholestatic	26 (40.6)	16 (41.0)	37 (27.6)	
Mixed	11 (17.2)	5 (12.8)	65 (48.5)	
Time to onset				0.78
5–90 days	34 (53.1)	21 (53.8)	60 (44.8)	
<5 or >90 days	16 (25.0)	10 (25.7)	39 (29.1)	
≤30 days from cessation of the drug	14 (21.9)	8 (20.5)	35 (26.1)	
Smoking	24 (37.5)	14 (35.9)	53 (39.6)	0.91
Alcohol abuse	23 (35.9)	17 (43.6)	54 (40.3)	0.72
Allergy history	19 (29.7)	11 (28.2)	28 (20.9)	0.34
Comorbidities				
Hypertension	20 (31.3)	13 (33.3)	41 (30.6)	0.95
Cardiovascular disease	10 (15.6)	1 (2.6)	19 (14.2)	0.11
Diabetes mellitus	12 (18.8)	2 (5.1)	16 (11.9)	0.12
Concomitant use of drugs				0.25
Concomitant drugs known as hepatotoxin	43 (67.2)	30 (77.0)	84 (62.7)	
Concomitant drugs with no hepatotoxin	21 (32.8)	9 (23.0)	50 (37.3)	

^1^ALT levels greater than 5 × the ULN and/or ALP levels greater than 2 × the ULN. ^2^ALT or AST levels greater than 5 × the ULN on two consecutive occasions and/or ALP levels greater than 2 × the ULN on two consecutive occasions. ^3^ALT levels greater than 1 × the ULN on two consecutive occasions or ALT levels greater than 2 × the ULN. RUCAM, Roussel Uclaf Causality Assessment Method; DILIN, DILI Network; NMPA, National Medical Products Administration.

The numbers of patients fulfilling Hy’s law in the three groups are listed in **Table 3**, and a significant difference was observed in each pairwise comparison (*P* < 0.01).

**Table 3:** The number of cases fulfilling Hy’s law in three groups of positive cases.

	RUCAM criteria^1^	DILIN criteria^2^	NMPA criteria^3^	*P* value
Hy’s law	16 (25.00%)	18 (46.15%)	12 (8.96%)	0.00

^1^ALT levels greater than 5 × the ULN and/or ALP levels greater than 2 × the ULN. ^2^ALT or AST levels greater than 5 × the ULN on two consecutive occasions and/or ALP levels greater than 2 × the ULN on two consecutive occasions. ^3^ALT levels greater than 1 × the ULN on two consecutive occasions or ALT levels greater than 2 × the ULN.

### Agreement Analysis Results

The agreement of the diagnostic results between the RUCAM criteria and the DILIN criteria is shown in **Table 4**. The number of patients with consistent results after the application of the two sets of criteria was 13,627, and the *Kappa* value was 0.50 (*P* < 0.01). Therefore, the level of agreement between the two sets of criteria was moderate.

**Table 4:** Agreement between the RUCAM criteria and DILIN criteria.

DILIN criteria^1^	RUCAM criteria^2^		Total
	Positive	Negative	
Positive	26	13	39
Negative	38	13,601	13,639
Total	64	13,614	13,678

^1^ALT or AST levels greater than 5 × the ULN on two consecutive occasions and/or ALP levels greater than 2 × the ULN on two consecutive occasions. ^2^ALT levels greater than 5 × the ULN and/or ALP levels greater than 2 × the ULN.

The agreement of the diagnostic results between the RUCAM criteria and the NMPA criteria is shown in **Table 5**. The number of patients with consistent results after the application of the two sets of criteria was 13,588, and the *Kappa* value was 0.54 (*P* < 0.01). Therefore, the level of agreement between the two sets of criteria was moderate.

**Table 5:** Agreement between the RUCAM criteria and NMPA criteria.

NMPA criteria^1^	RUCAM criteria^2^		Total
	Positive	Negative	
Positive	54	80	134
Negative	10	13,534	13,544
Total	64	13,614	13,678

^1^ALT levels greater than 1 × the ULN on two consecutive occasions or ALT levels greater than 2 × the ULN. ^2^ALT levels greater than 5 × the ULN and/or ALP levels greater than 2 × the ULN.

The agreement of the diagnostic results between the DILIN criteria and the NMPA criteria is shown in **Table 6**. The number of patients with consistent results after the application of the two sets of criteria was 13,559, and the *Kappa* value was 0.31 (*P* < 0.01). Therefore, the level of agreement between the two sets of criteria was poor.

**Table 6:** Agreement between the DILIN criteria and NMPA criteria.

NMPA criteria^1^	DILIN criteria^2^		Total
	Positive	Negative	
Positive	27	107	134
Negative	12	13,532	13,544
Total	39	13,639	13,678

^1^ALT levels greater than 1 × the ULN on two consecutive occasions or ALT levels greater than 2 × the ULN. ^2^ALT or AST levels greater than 5 × the ULN on two consecutive occasions and/or ALP levels greater than 2 × the ULN on two consecutive occasions.

## Discussion

In recent years, reports on DILI have rapidly increased in number, and research on the underlying mechanism is becoming increasingly in-depth. Unfortunately, due to its low incidence in the population, preclinical testing in animal models and *in vitro* toxicology systems have provided minimal assistance in identifying potentially hepatotoxic drugs, and DILI remains one of the most challenging disorders faced by gastroenterologists (Fontana et al., 2009; Chalasani et al., 2014). Thus, establishing standardized DILI diagnostic criteria is one of the challenges that must be overcome. To date, many research projects and institutions have published a variety of liver test criteria for DILI. This study selected the RUCAM criteria as the gold standard and compared them with the DILIN criteria and NMPA criteria. The aims were to determine if these alternative criteria are also suitable for detecting DILI and to provide reliable evidence for the best set of criteria to use in research or clinical practice.

In mainland China, as indicated in many studies, traditional Chinese medicine (TCM) was frequently reported for hepatotoxicity and has been increasingly recognized (Teschke et al., 2016; Zhu et al., 2016; Jing and Teschke, 2018; Wang et al., 2018; Shen et al., 2019). As is well known, TCM, which includes natural medicines, Tibetan medicines, and Mongolian medicines, is being used increasingly worldwide, especially in China due to the historical background of TCM use, and it is the major offending agent of DILI (Shen et al., 2019). However, a large proportion of TCM was used mostly among outpatients, and they were only admitted to the hospitals while experiencing or being diagnosed with DILI. It was difficult to proactively monitor the occurrence of TCM-related liver injury among outpatient populations. In addition, compared with western medicine, the causality assessment of liver injury caused by TCM was more difficult, and there were limitations in applying the RUCAM in DILI caused by TCM (Zhu et al., 2016). Thus, we did not choose TCM as our target drug in this study.

Of these three sets of criteria, the DILIN criteria are the most stringent. Elevated aminotransferase or ALP levels must be detected in two successive measurements, and elevated AST levels are also considered. The RUCAM criteria are the second most stringent and only require an observation of increased ALT and ALP levels in one assessment. In contrast, the NMPA criteria are much less stringent.

According to the results of active monitoring by the ADE-ASAS and manual assessment, some of the possible cases (total score of 3–5 points) might not be DILI and could be attributed to other causes, such as tumors or sepsis. Therefore, these patients were not included as positive cases to ensure the reliability of the data and results. Overall, a significant difference in PPV was not observed, and the PPV was very low for all three sets of criteria because hepatotoxicity diagnoses currently must include other necessary examinations and estimates of causality by pharmacists to exclude alternative causes in addition to laboratory tests. Therefore, biomarkers that accurately and efficiently diagnose DILI are urgently needed.

Based on the results of the *Kappa* statistic, the level of agreement between the RUCAM criteria and the other two sets of criteria were moderate, and the level of agreement between the DILIN criteria and the NMPA criteria was poor. The results based on criteria that require increased levels of liver enzymes in two consecutive measurements or lower thresholds of these parameters still have certain consistency with the gold standard. However, the generalizability of the study results, with respect to other potentially causative agents of DILI, is limited because only patients with suspected fluoroquinolone-induced liver injury were included.

Further analysis showed that compared with the RUCAM group, the DILIN group exhibited 299 fewer warnings and 25 fewer positive cases, and the NMPA group had 720 more warnings and only 70 more positive cases. The manpower required to evaluate the warnings returned by the application of the NMPA criteria was substantially more than that required to evaluate the warnings returned by the application of the RUCAM criteria, whereas the number of confirmed positive cases increased much less. In contrast, the DILIN criteria are more efficient and less labor-intensive than the RUCAM criteria, but also missed 25 cases. As the NMPA criteria are the least strict, most of the positive cases were self-limited liver injury, which can completely recover without much treatment. If DILI is diagnosed according to this set of criteria, clinicians will spend more time evaluating cases that do not need their attention.

Moreover, Hyman Zimmerman, a pioneer of modern hepatotoxicology, proposed that the combination of jaundice and drug-induced hepatocellular injury was associated with a 10% to 50% mortality rate for liver failure after other causes for increased bilirubin levels were excluded, which has been referred to as “Hy’s Law” (Robles-Diaz et al., 2014). As shown in **Table 3**, the proportions of patients diagnosed using the RUCAM and DILIN criteria who fulfilled Hy’s Law were significantly greater than the proportion diagnosed using the NMPA criteria, and the DILIN group had the highest proportion of patients fulfilling Hy’s law. This further demonstrated that much stricter criteria effectively eliminated the low-risk population and helped clinicians focus their limited energy on high-risk patients.

However, the DILIN criteria also have some limitations. Because of the inclusion of stricter indicators, patients who are confirmed to be experiencing DILI usually present with a serious and complicated condition, and the course of the disease is prolonged. Clinicians are unable to easily relate liver injury to the drugs used. For instance, in the present study, only six (15.38%) cases in the DILIN group were clearly diagnosed with “drug-induced liver injury” or “liver injury related to drugs” in the medical records, compared with 34 (53.13%) in the RUCAM group. Therefore, we believe that the RUCAM criteria is relatively more suitable for clinical practice.

## Conclusion

In this study, the NMPA criteria, with relatively lax thresholds of parameters, require extensive labor to determine a diagnosis, making them unsuitable for clinical practice. Conversely, the level of agreement between the RUCAM criteria and the DILIN criteria was moderate, and the diagnostic results achieved with these criteria are consistent with each other to a certain extent. The DILIN criteria, with stricter thresholds for the parameters, are more effective and might help clinicians focus their attention on seriously ill patients. However, this set of criteria may miss some positive cases, and the patients identified usually presented with quite serious conditions, which are not conductive to early interventions. Therefore, we still recommend the use of the RUCAM criteria in clinical practice.

## Data Availability

All datasets generated for this study are included in the manuscript and the supplementary files.

## Author Contributions

All coauthors are justifiably credited with authorship, according to the authorship criteria. Final approval was given by each coauthor. HY: conception, design, data collection, analysis of data, interpretation of results, drafting, and revision of manuscript. DG: conception, design, data collection, analysis of data, interpretation of results and critical revision of manuscript. YX, MZ, and CY: analysis of data, interpretation of results. CC and WJ: revision of manuscript.

## Funding

This study was supported by the National Natural Science Foundation of China (grant 81773825).

## Conflict of Interest Statement

The authors declare that the research was conducted in the absence of any commercial or financial relationships that could be construed as a potential conflict of interest.
